# Unveiling the Perspectives on Sexual Unwellness: A Cross-National Qualitative Study with Older Adults from Mexico and Portugal

**DOI:** 10.1192/j.eurpsy.2024.435

**Published:** 2024-08-27

**Authors:** S. von Humboldt, N. M. Mendoza-Ruvalcaba, J. A. Ribeiro-Gonçalves, A. Chávez-Rodríguez, E. D. Arias-Merino, G. Low, I. Leal

**Affiliations:** ^1^William James Center for Research, ISPA – Instituto Universitário, Lisbon, Portugal; ^2^University of Guadalajara CUTONALA, Health Sciences Division, Guadalajara, Jalisco; ^3^Public Health Department, Universidad de Guadalajara CUCS, Guadalajara, Mexico; ^4^Faculty of Nursing, University of Alberta, Edmonton, AB, Canada

**Keywords:** Cross-cultural, Older adults, Qualitative study, Sexual Unwellness

## Abstract

**Introduction:**

Sexual unwellness (SU) has been linked to a lack of sexual satisfaction and to an incapacity to maintain sexual relations.

**Objectives:**

The objective of this cross-cultural study is to shed light on older adults’ perspectives on SU across cultures.

**Methods:**

Eighty-three older participants (65 to 98 years of age) took part in this qualitative study. Participants lived in the community and were of two different nationalities (Portuguese and Mexican). Semi-structured interviews were conducted, and content analysis was then carried out.

**Results:**

SU emerged in the findings of the content analysis through six themes: Dissatisfying Sexual Experiences; Feelings of Isolation; Spirituality; Medication; Unattractiveness and Pain. The most common theme among older Portuguese participants was ‘Dissatisfying Sexual Experiences’ (25.5%). ‘Feelings of Isolation’ was most common among older Mexican participants (13.7%).

**Conclusions:**

A diversity of experiences of older adults in relation to SU was highlighted in this study. Moreover, cross-cultural research on the construct of SU is essential for understanding the cultural differences in the conceptualization of the construct and how these themes may influence the quality of sexual life in old age.

**Disclosure of Interest:**

None Declared

